# A Novel Field-Circuit FEM Modeling and Channel Gain Estimation for Galvanic Coupling Real IBC Measurements

**DOI:** 10.3390/s16040471

**Published:** 2016-04-02

**Authors:** Yue-Ming Gao, Zhu-Mei Wu, Sio-Hang Pun, Peng-Un Mak, Mang-I Vai, Min Du

**Affiliations:** 1 College of Physics and Information Engineering, Fuzhou University, Fuzhou 350116, China; 15280106863@163.com (Z.-M.W.); dm_dj90@163.com (M.D.); 2 Key Lab of Medical Instrumentation & Pharmaceutical Technology of Fujian Province, Fuzhou 350116, China; fstpum@umac.mo (P.-U.M.); fstmiv@umac.mo (M.-I.V.); 3 State Key Laboratory of Analog and Mixed Signal VLSI, University of Macau, Macau 999078, China; lodge@mail.eee.umac.mo; 4 Department of Electrical and Computer Engineering, Faculty of Science and Technology, University of Macau, Macau 999078, China

**Keywords:** galvanic coupling intra-body communication, field-circuit coupling, channel modeling, channel estimation

## Abstract

Existing research on human channel modeling of galvanic coupling intra-body communication (IBC) is primarily focused on the human body itself. Although galvanic coupling IBC is less disturbed by external influences during signal transmission, there are inevitable factors in real measurement scenarios such as the parasitic impedance of electrodes, impedance matching of the transceiver, *etc.* which might lead to deviations between the human model and the *in vivo* measurements. This paper proposes a field-circuit finite element method (FEM) model of galvanic coupling IBC in a real measurement environment to estimate the human channel gain. First an anisotropic concentric cylinder model of the electric field intra-body communication for human limbs was developed based on the galvanic method. Then the electric field model was combined with several impedance elements, which were equivalent in terms of parasitic impedance of the electrodes, input and output impedance of the transceiver, establishing a field-circuit FEM model. The results indicated that a circuit module equivalent to external factors can be added to the field-circuit model, which makes this model more complete, and the estimations based on the proposed field-circuit are in better agreement with the corresponding measurement results.

## 1. Introduction

With the great improvement of science and technology, there has been a boom in wearable devices whose applications include sports, wearable entertainment devices and biomedical devices. In the coming future, wearable devices will not be limited to smart watches, smart glasses or other consumer electronic products under the trend of Internet plus mobile health. Certainly, with higher design requirements in reliability, accuracy and low power consumption, medical wearable devices play a prominent role [1,2,3]. Human-centered is the major characteristic of wearable devices. In this regard, how to realize the communication of wearable devices in the human body environment is of great importance, especially when it relates to implantable devices. Intra-body communication (IBC) uses the human body as the transmission medium for electrical signals [4]. Due to the conductivity of the human tissues, IBC has the desirable characteristics of low power consumption, convenient connection, no need for antenna design, and high security [5], which make it particularly suitable for communication between wearable devices, especially implantable wearable devices.

Based on the different coupling types, intra-body communication can be divided into capacitive coupling and galvanic coupling. In capacitive coupling, the signal is injected into the human body through a signal electrode at the transmitter and detected by a signal electrode at the receiver, and the return path consists of the earth electrodes and external environment [4]. In galvanic coupling, the electrical current signal is transmitted through transmitter electrodes to the receiver electrodes via the human body [6].

The special characteristics of the human channel play a vital role in the study of IBC. Thus previous research mostly focused on the human channel with a numerical simulation method, an analysis method or an equivalent circuit in order to clarify the signal transmission mechanism. For the capacitive-type IBC, since the frequency is higher than 10 MHz, signal transmission is considered to occur mainly through surface waves [7]. An finite difference time domain (FDTD) method was considered to be the most suitable method for studying the transmission mechanism of the intra-body communication [8]. Fujii *et al.* used the FDTD method to simulate the transmission of electrical signals in the arm, reaching the conclusion that a simple uniform model can be used to model the transmission mechanism of capacitive coupling IBC. Bae *et al.* [7] proposed a numerical model of capacitive coupling IBC; their model consisted of a quasi-static electric field, the induction field and surface wave field. Of course, a lot of research teams have used the circuit model to create a body channel equivalent. Pereira *et al.* [9] proposed a mixed distributed lumped model to express the channel and text fixtures, and divide the channel response into two parts: internal and external. Xu *et al.* [10] adopted a finite element method (FEM) to simulate the received signals, the coupling between the human body and earth were equivalent to a parallel RC model.

The signal in the galvanic coupling type is within the body as it is transmitted from the transmitter to the receiver. Therefore, it is less affected by the external environment. This gives it more reliability in the medical or healthcare field. Our work here concentrates on the galvanic-type IBC because of its security and less interference features. Swaminnathan *et al.* [11] analyzed the channel through tissue layers, which can be equal to a 3-D circuit model. Kibret *et al.* [12] calculated the electrode-skin impedance in a a new way and generated an equivalent upper arm as a circuit model. A complete transfer function was obtained on the basis of an improved four terminal circuit model by Song *et al.* [13]. Pun *et al.* [14] came up with an analytical quasi-static electromagnetic model for the IBC signal distribution in the human upper arm. Based on this model, Chen *et al.* [15] obtained a more complete human body transfer function. However, almost all of the above models abstracted the human body as a regular geometric object. Research has yet to consider the effect of people’s anatomical characteristics on signal transmission. According to its anatomical characteristics, Callejon *et al.* [16] equated the human body upper arm including skin, fat, muscle, cortical bone and cancellous bone, to a five layer concentric cylinder model using FEM. Zeng *et al.* [17] proposed a four layer concentric cylinder model in an analysis of the effect of muscle anisotropy on signal transmission.

The finite element method can reconstruct the distribution of the internal potential and current density caused by the transmission signal, and realize a visualization of the signal transmission. However, the electrodes of an IBC transceiver are in direct galvanic contact with the human body, which is an ionic conductor [18], so when the electronic current is converted into an ion current by the transmitter electrodes, and the receiver electrodes transform the ion current into an electronic current, some electrochemical reactions occur at the transceiver electrodes. Therefore, a parasitic impedance between the electrodes will be generated when the frequency is high enough [18]. In practice, IBC systems measure the parasitic impedance between the electrodes, and the input and output impedances of testing instruments in addition to the human channel proper. For this reason, the complete IBC system consists of the electrodes, signal source and a voltmeter [18]. These factors are often overlooked when using the FEM. To avoid these problems, this paper will establish a field-circuit FEM model of a galvanic coupling IBC within an actual measurement environment, to realize the channel gain estimates based on a field-circuit model.

The model incorporates parasitic impedance between the electrodes, signal source, and voltmeter into a circuit model, combined with an anisotropic arm model, establishing a field-circuit FEM model of a galvanic coupling IBC. The channel is divided into a short part and a long part according to the channel characteristics of the human arm, and on the basis of the model realize channel estimations.

The arrangement of this paper is as follows: Section 2 focuses on the field-circuit FEM modeling, establishing the model in a practical measurement environment. Short distance and long distance channel characteristics estimation are presented in Section 3. Section 4 mainly verifies the feasibility and the accuracy of the channel characteristic estimations by using *in vivo* measurements. Section 5 concludes the paper.

## 2. Field-Circuit FEM Modeling

### 2.1. Electric Field Modeling

**Figure 1:**
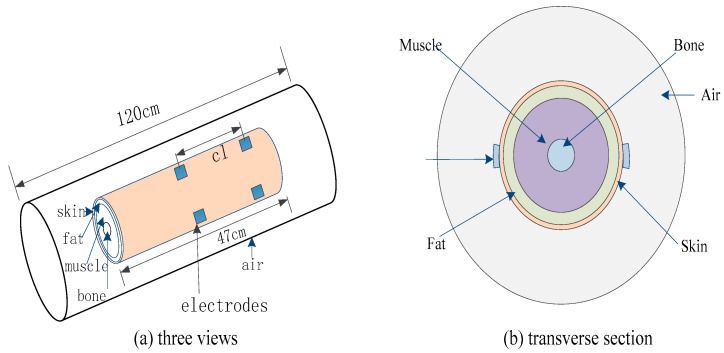
The electric field model of the signal transmission path in the galvanic intra-body communication. (**a**) Three dimensional view of the electric field model; (**b**) Cross section of the electric field model.

Based on our previous work [14], the human upper limb was also approximated to a concentric cylinder with four layers, consisting of bone (12.5 mm), muscle (27.5 mm), fat (8.5 mm), and skin (1.5 mm). The transmitter and the receiver each have a pair of electrodes, as shown in Figure 1. The solution of the model was calculated based on the finite element method found in COMSOL Multiphysics 5.0 (COMSOL Inc., Stockholm, Sweden) . It is important to note that the air layer was considered, in order to simulate the infinite domain surrounding the upper limb model.

In galvanic coupling IBC, the total charge density is zero within the model, and human tissue is simulated with the quasi-static field approximation condition at low frequency. Maxwell's equation is simplified to the Laplace equation [14,19,20]:

(1) $$-\nabla \times \left[\left(\sigma +i\omega \varepsilon_{0}\varepsilon_{i}\right)\nabla V\right]=0$$

where σ represents the electrical conductivity, ω represents the angular frequency, ε_0_ represents the permittivity of the vacuum, ε_*i*_ represents the relative permittivity and *V* represents the scalar electric potential.

The dielectric properties of human tissues, such as conductivity and permittivity, were derived from the parametric modes of Gabriel [21]. Muscle fibers are excited by an external excitation source, depolarized, and a current is generated, which then spreads to the whole volume of the human body. However, the propagation velocity of the current in the muscle layer, differs according to the direction. Muscle conductivity differs by directions, caused by the arrangement of muscle fiber. Current tends to propagate along the axial direction of the muscle fiber. For this reason, the muscle conductivity is set as anisotropic [17]. In our previous study results [17] it can be found that when considering the anisotropy of the muscle tissue, the results of an electric field (EF, a list of acronyms and their meaning as shown in Table 1.) model will be closer to the human body experimental results. At the same time, the anisotropy property of muscle tissue is an inherent attribute, so we think it is reasonable to consider this property. The conductivity of tissues used in our model is shown in Table 2.

Input electrical signal at the transmitter electrodes:

(2) $$V=V_{0}$$

where *V*_0_ is the voltage amplitude injected into the limb.

The voltage and current continuous conditions were established at the interface between the adjacent layers:

(3) $$\left\{ \begin{array}{l} V_{l-1}=V_{l}\\ J_{l-1}=J_{l} \end{array}\right.$$

*J*_*l*−1_, *J_l_* and *V*_*l*−1_, *V_l_* indicate the current density and the voltage of the adjacent tissues, and *l* indicates the tissue layer, *l* = 4, 3, 2.

Current and voltage continuity conditions of the receiving electrodes and of the surface of the human model are given by:

(4) $$\left\{ \begin{array}{l} V_{l}=V_{r}\\ J_{l}=J_{r} \end{array}\right.$$

where, *V_r_* and *J_r_* represent the voltage and the current density of the receiving electrode.

Thus, a default fine free tetrahedral element is used to mesh the arm geometry. The solution for the model is obtained by running the direct solver PARDISO in a fully coupled manner. The degrees of freedom are 38,365.

### 2.2. Field-Circuit Modeling

#### 2.2.1. Field-Circuit Model

In the *in vivo* measurement, the signal source generates electrical signals, and transmits them through the transmitter electrodes into the body, where they are received differentially by two receiver electrodes. All electrodes are in direct galvanic contact with the human body, which is an ionic conductor. Therefore the parasitic impedance between the electrodes may be intolerably high when the frequency is high enough (more than 5 kHz) [18]. At the same time, variations in the value of the impedance affect the potential difference, which depends on the material of the electrodes, and the distance between transceiver electrodes.

In practice, the signal source generates an electrical signal, which is injected into the human body and detected by the voltmeter. The energy exchange between the apparatus and human body changes the working state of the measured object to a certain extent [22]. The internal impedances of the generators and voltmeters will affect the measurement accuracy. It should ensure that the load impedance and the internal impedance of the signal source be matched each other, to get the maximum power output of work status. We should try to increase the input impedance of the voltmeter, weakening the load effect, to reduce the measurement error. Therefore, in the process of IBC modeling, the parasitic impedance resulting from the electrodes as well as the measurement system parameters (source and voltmeter impedances) should be considered [18].

**Figure 2:**
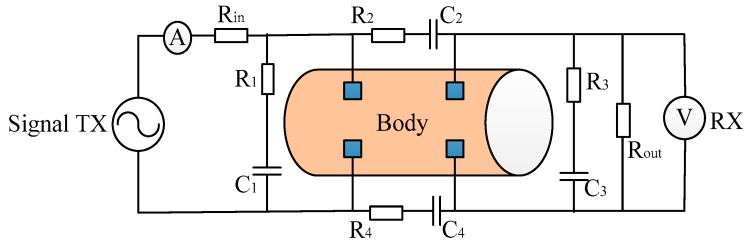
The field-circuit model of signal transmission path in the galvanic intra-body communication.

The *in vivo* measurement scene in this paper can be equivalent to the field-circuit model shown in Figure 2, where, *R*_1_–*R*_4_, *C*_1_–*C*_4_ represent the parasitic impedance, and *R*_in_ and *R*_out_ are the input and output resistance of the transmitter and receiver. This uses four electrodes of the electric field model to realize the combination of electric module and circuit module found in COMSOL Multiphysics 5.0.

The Laplace equation and boundary conditions of electric field module were consistent with the electric field model. For the sake of realizing the signal transmission from an external circuit to the arm, the circuit module was provided with four External I Terminal nodes, and the four electrodes were chosen as Terminal nodes.

#### 2.2.2. Field-Circuit Model Parameter Estimation

**Figure 3:**
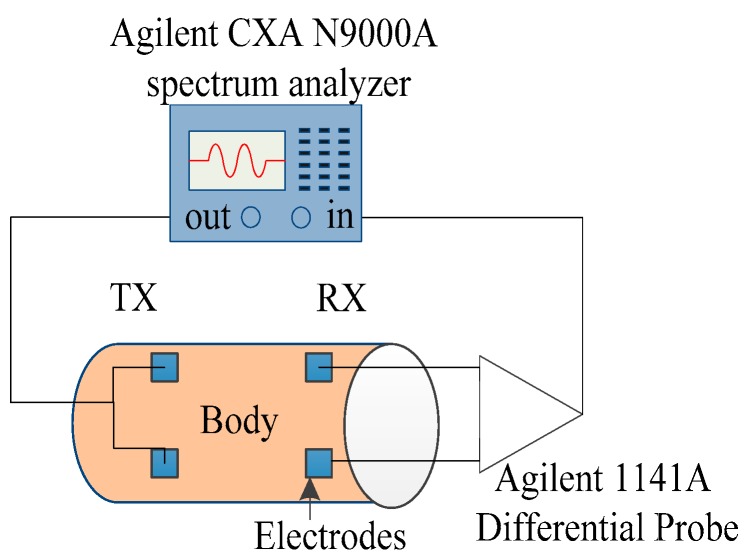
Experimental setup for the body channel measurement, Agilent CXA N9000A spectrum analyzer as a transmitter, Agilent 1141A differential probe and spectrum analyzer as a receiver.

In order to determine the parameter values, a galvanic coupling IBC experiment was carried out on a human arm. We opted for a spectrum analyzer (CXA N9000A, Agilent, Santa Clara, CA, USA), with one port as the transmitter and the other port as the receiver. Its experimental block diagram is given in Figure 3.

In this way, the power of the injected signal was 0 dBm. A differential probe was adopted to detect the received signal so that the IBC ground from the equipment grounds could be decoupled. The received signal in the form of voltage gain is displayed on the screen. The noise floor of the spectrum analyzer Agilent CXA N9000A is −120 dB, so we do not need to worry about being limited by the dynamic range of the spectrum analyzer. The voltage gain is used to represent the channel attenuation characteristics, with their expression shown in Equation (5), *V_r_* being the receiving voltage, *V_t_* being the transmitting voltage:

(5) $$Gain\left(\text{cl}\right)=20\cdot \text{log}\left(V_{r}/V_{t}\right)$$

**Figure 4:**
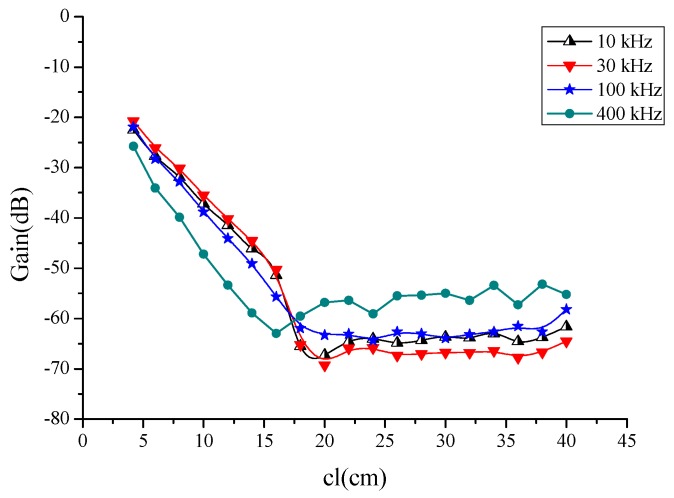
Mean values of the intra-body communication channel characteristics, measured on six test subjects at different frequencies over a period of several days.

It should be noted that the measurements were repeated over a period of time with six test subjects to verify the accuracy and rigor of the experiment. The results were consistent over a period of time, and the average data is reported in Figure 4.

**Figure 5:**
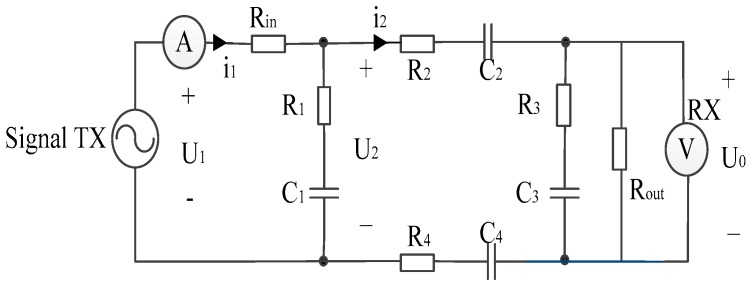
Equivalent circuit model of the circuit module in the field-circuit model, including the parasitic impedance between the transceiver electrodes, the internal impedances of the voltmeters and generators.

As shown in Figure 4, the voltage gain was dependent on the signal channel length cl, so we fixed the *R*_1_–*R*_4_, *C*_1_, *C*_3_, and let *C*_2_ = *C*_4_, to indicate the change in the channel length. In this paper, *R*_in_ = 50 Ω and *R_out_* = 1 MΩ, parasitic impedance has a decreasing characteristic with frequency, *R*_1_ = *R*_3_ = 330 Ω at 10 kHz and a value of 101 Ω at 1 MHz. Meanwhile, the *C*_1_ = *C*_3_ = 42 nf at 10 kHz and a value of 13.6 nf at 1 MHz. In COMSOL Multiphysics, the EF model was satisfied with the Maxwell’s Equations, and the circuit model abides the Kirchhoff’s law. They were computed simultaneously in the software. In the C-EF model here, the excited signal of the EF part was decided by the source of the circuit, which was usually regarded as a constant of current or voltage. It led to the electric current signal volume in the EF model maintaining the same propagation for each simulation. Thus, the parts of the EF and circuit could be treated as decoupled in this paper. When we paid attention to the relationship of the signal attenuation and the channel length of the human body, the parasitic impedance between the transceiver electrodes remained as the only different parameter in the field-circuit (C-EF) model., so in the next step, the equivalent circuit model was analyzed in Figure 5.

In the circuit model as shown in Figure 5, we require:

(6) $$\{\begin{array}{c} \begin{array}{cc} Z_{1}=\frac{1}{j\omega C_{1}}+R_{1} & Z_{2}=\frac{1}{j\omega C_{2}}+R_{2} \end{array}\\ \begin{array}{cc} Z_{3}=\frac{1}{j\omega C_{3}}+R_{3} & Z_{4}=\frac{1}{j\omega C_{4}}+R_{4} \end{array}\\ \begin{array}{cc} R_{2}=R_{4} & C_{2}=C_{4} \end{array} \end{array}$$

(7) $$Z_{out1}=\frac{Z_{3}\cdot R_{out}}{Z_{3}+R_{out}}$$

(8) $$Z_{out}=Z_{2}+Z_{out1}+Z_{4}$$

According to the Kirchhoff’s voltage law and Kirchhoff’s current law:

(9) $$U_{2}=i_{2}\cdot Z_{\text{out}}$$

(10) $$U_{0}=i_{2}\cdot Z_{out1}$$

(11) $$U_{1}=i_{1}\cdot (R_{\text{in}}+\frac{Z_{1}\cdot Z_{out}}{Z_{1}+Z_{out}})$$

(12) $$U_{2}=(i_{1}-i_{2})\cdot Z_{1}$$

By Equations (6)–(12), the transfer function of the circuit model is:

(13) $$g=\frac{U_{0}}{U_{1}}=\frac{Z_{out1}\cdot Z_{1}}{Z_{1}+Z_{2}+Z_{4}+Z_{out1}}÷(R_{in}+\frac{Z_{1}\cdot Z_{out}}{Z_{1}+Z_{out}})$$

The relationship between the channel characteristics and the transfer function g was as follows:

(14) G(cl)=20⋅log10(g)

which can be obtained by Equation (13):

(15) $$C_{2}=Y(C_{2})=\frac{2\cdot g\cdot Z_{1}+2\cdot g\cdot R_{in}}{j\cdot \omega \cdot [Z_{out1}\cdot Z_{1}-g\cdot R_{in}\cdot Z_{1}-g\cdot Z_{1}\cdot Z_{out1}-Z_{out1}\cdot g\cdot R_{in}-2\cdot g\cdot Z_{1}\cdot R_{2}-2\cdot g\cdot R_{in}\cdot R_{2}]}$$

## 3. Channel Estimation

From Figure 4, we can see attenuation increases gradually as the channel length increases from 4 cm up to 20 cm and remains approximately constant for longer channels. The difference between frequencies is approximately constant. Thence, the channel can be divided into a short channel section (when cl ≤ 20 cm, the absolute attenuation value increased as the channel length increased) and a long channel section (when cl ≥ 20 cm, the absolute attenuation value is approximately constant).

### 3.1. Short Channel Gain Estimated

**Figure 6:**
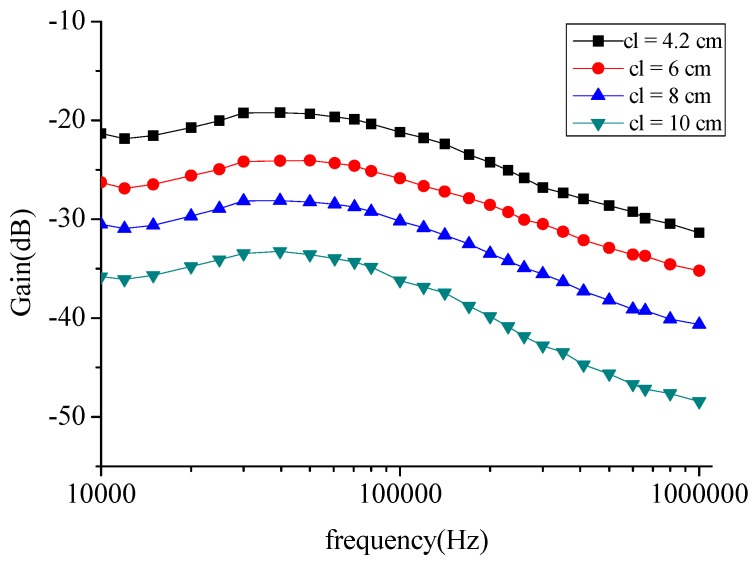
Circuit model channel characteristics for different distances, here the part of the EF and circuit were treated as decoupling in C-EF model.

Attenuation increases gradually as the channel length increases from 4 cm to 20 cm, where attenuation depends strongly on the channel length. Within the short channel section, the channel characteristics of the circuit model of Figure 5 are as shown in Figure 6.

The trends observed for all channel lengths were quite similar, just as the length of the channel increases by 2 cm, the attenuation value increases by about 5 dB. It can be seen that the channel gain is dependent on the channel length, so the longer the channel length, the greater the voltage attenuation. Consequently, the characteristic curve of the channel length is at 4.2 cm as a baseline *G*(bl). A function of the channel length *K*(cl) can be obtained by dividing the baseline *G*(bl) into the other channel characteristic, *G*(cl):

(16) $$K(cl)\approx \frac{G(cl)}{G(bl)}$$

Table 3 representative K(cl) at different distances. The study found that *K*(cl) is a one-dimensional linear function dependent upon channel length.

**Figure 7:**
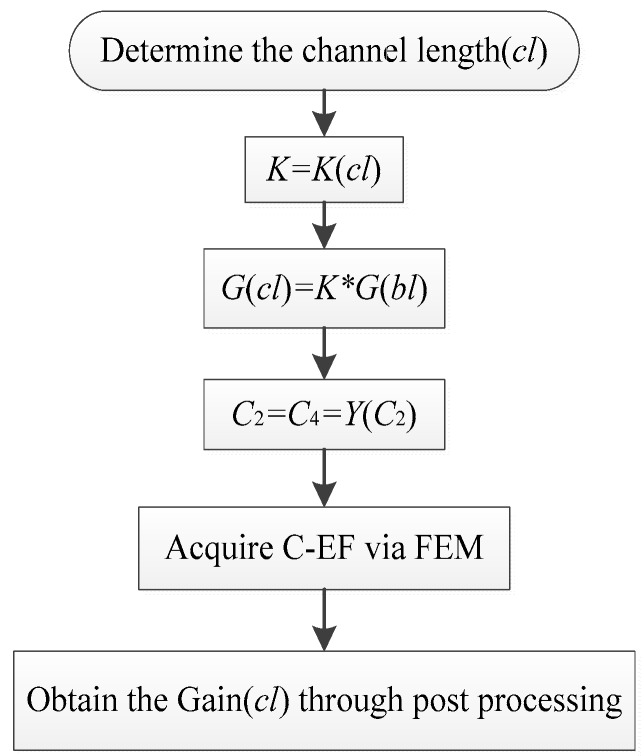
The flow chart of the short distance intra-body communication channel characteristics estimated in Field-Circuit model.

*K* can be obtained by examining the function of *K*(cl) after determining the channel length and applying Equation (15) to obtain the channel characteristics *G*(cl) of the circuit model. The relationship between capacitance *C*_2_ and channel characteristics *G*(cl) is used to solve for *C*_2_. Inserting the capacitance value into the field-circuit model, the channel characteristics can be estimated. The flow chart as shown in Figure 7.

### 3.2. Long Channel Gain Estimated

**Figure 8:**
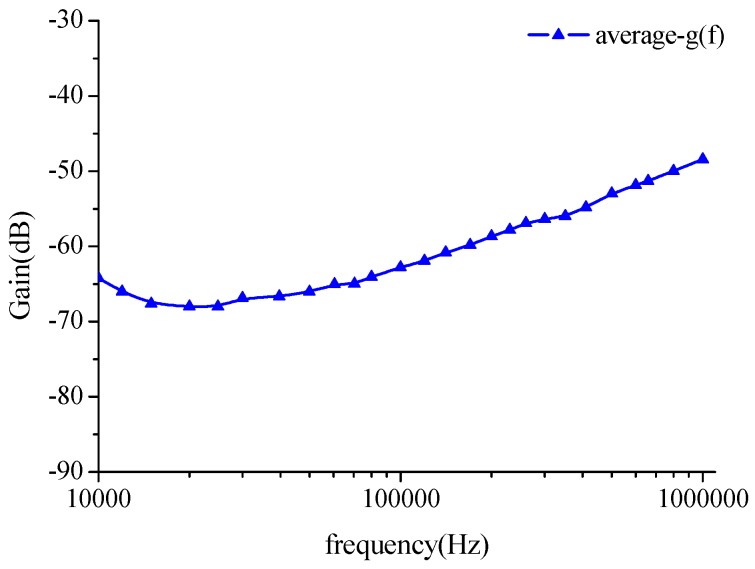
Mean values of the estimation results for long distance intra-body communication channel characteristics in the field-circuit model.

Voltage attenuation and channel length are seem to be independent of each other within the range of 20 cm to 40 cm, as the channel length continues to increase, the attenuation remains substantially. Furthermore, after selecting the average attenuation value within the scope as the basic characteristic of the channel, *g*(*f*) the average curve *g*(*f*) in Figure 8 represents the basic characteristic of long channels.

Making *G* = *g*(*f*) + *M* denotes the channel characteristics at long lengths, where *M* is the correction factor used to correct the gap between measurements and the simulations. Generally, *M* is influenced by the individual differences, the measurement precision, the environmental influence, *etc*.

### 3.3. Channel Gain Estimated Expression

To sum up, from the field-circuit (C-EF) model, we can obtain the human channel attenuation function expression:

(17) $$H_{dB}(f)=20\cdot {\text{log}}_{10}(V_{r}/V_{t})=\{\begin{array}{cc} \left|AH(f,\text{cl})\right|e^{j[\varphi (f)+\phi (\text{cl})]} & \text{cl}\leq 20\;\text{cm}\\ \left|AH(f)\right|e^{j[\varphi (f)]} & \text{cl}\geq 20\;\text{cm} \end{array}$$

When the channel length is less than 20 cm, the channel attenuation *H_dB_*(*f*) is dependent on the signal frequency *f* and channel length cl. When the channel length is greater than 20 cm, the attenuation depends only on signal frequency *f*, regardless of channel length cl.

## 4. The Measurement Experiments

To verify the feasibility and the accuracy of the channel characteristic estimations, an experiment was carried out on the other 10 people’s two hands. The experimental steps are as same as Figure 3.

**Figure 9:**
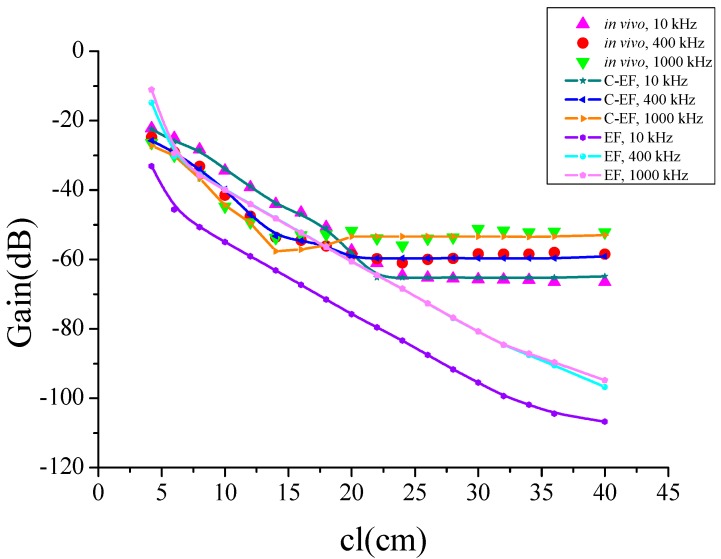
Mean values of the intra-body communication channel characteristics for the field-circuit model, electric field model and the body experiments, measured on 10 test subjects at different frequencies.

At the same time, to demonstrate the necessity of the field-circuit model, the results of the electric field (EF) model and channel estimation results of field-circuit (C-EF) model were compared with the experimental results in Figure 9. For the C-EF model, the simulation results are in good agreement with the experimental results, as their general tendency of gain *versus* channel length is same. Both increase with the increase of the channel length, and then remain unchanged after a certain value. At the same time, the absolute values of the errors between the estimation and the measurement are limited to 3 dB. On the other hand, there are a very small variation in the numerical value of the channel curve at different frequencies. For the EF model, the absolute attenuation value increased as the channel length increased from 4 cm to 20 cm, basically agreeing with the corresponding measurement results, but as the channel length increased from 20 cm to 40 cm, the attenuation values continued to rise, and there was thus a big deviation with the experimental results. This demonstrates the necessity of using the field-circuit model.

### 4.1. Short Channel Estimation Results

**Figure 10:**
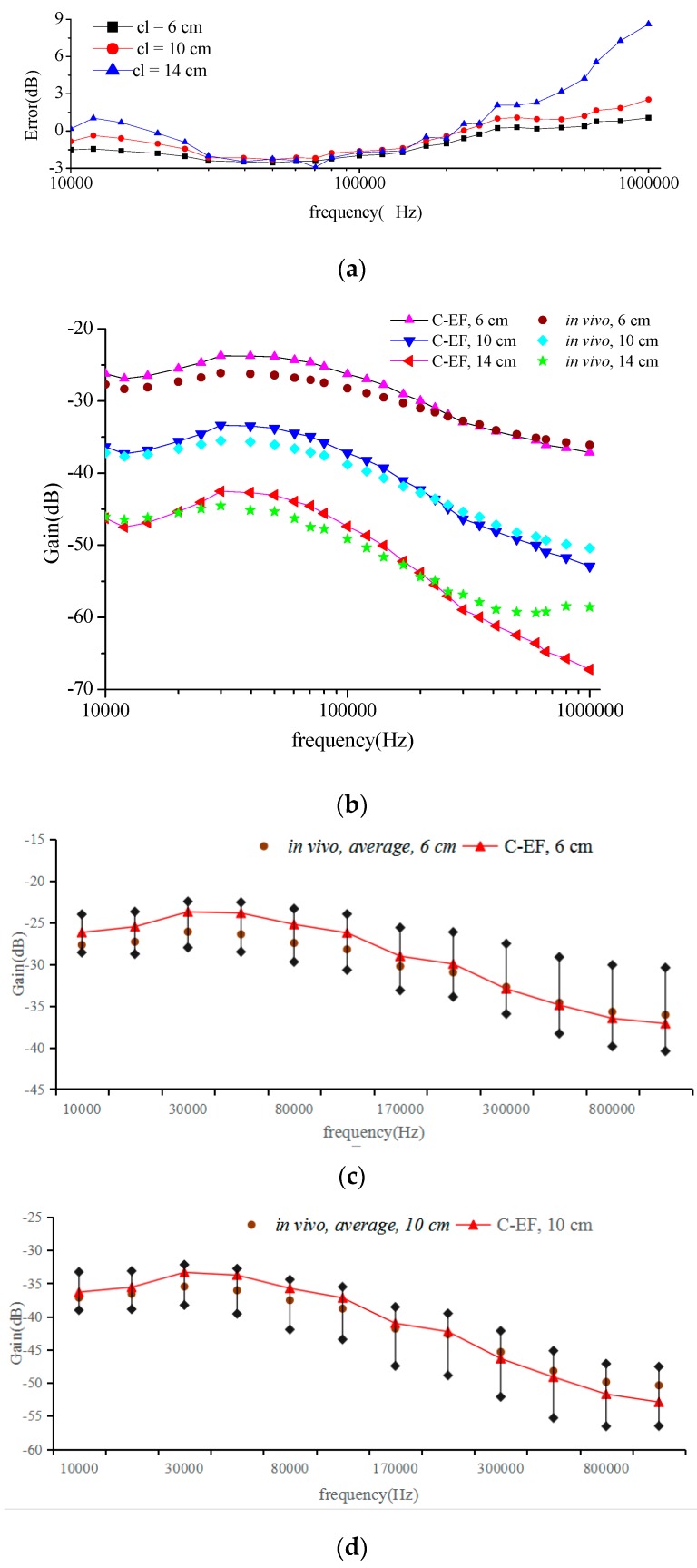

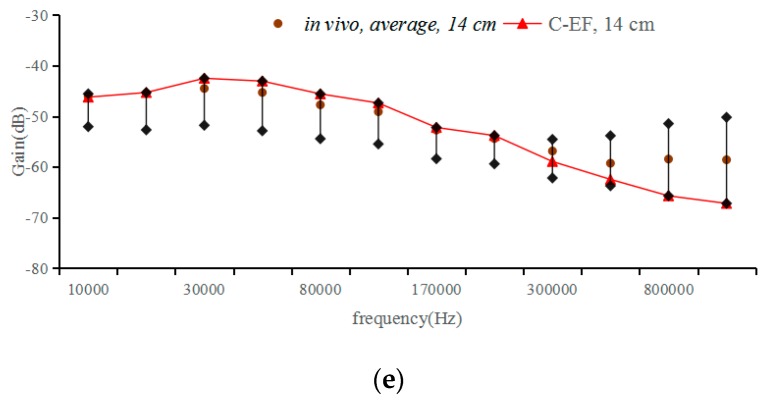
Comparison between *in vivo* measurements and C-EF channel estimation results, measured on 10 test subjects at different distances, (**a**) error of the experiment and simulation results, (**b**) the results of experiment and simulation; (**c**) results under 6 cm; (**d**) results under 10 cm; (**e**) results under 14 cm.

Figure 10 shows the comparison of the *in vivo* tests and the estimation results based on the flow chart in Figure 7. Including the voltage gain of the channel length 6 cm, 10 cm, 14 cm and the errors between the measurement and the estimation.

The estimation results and average values of tests under 6 cm, 10 cm and 14 cm are can be seen in Figure 10b and there are errors in Figure 10a, and Figure 10c–e reflecting the variation of gain among measurements of channel length under 6 cm, 10 cm, and 14 cm respectively. Black diamond representing the variation range of the measurements. It can be seen that the results of channel estimation and the corresponding experimental results agree to a very high degree. Meanwhile, the trend of the estimation results shows better agreement with the measured result in the low frequency range than in the high frequency range when the channel length is more than 14 cm. The error between measurement results and estimation results are less than 3 dB in the range 10 kHz to 500 kHz, and less than 9 dB in the range 500 kHz to 1 MHz. The error between measurement results and estimation results is within an acceptable range.

A larger deviation occurs from 500 kHz when the channel length is more than 14 cm. This is because the attenuation of the baseline *G*(bl) continues to increase at these higher frequencies, while *K*(cl) is a constant independent of the frequency. We can use the Equation (16) to estimate the channel characteristics *G*(bl), and *G*(bl) also continues to increase at these higher frequencies. However, the measurement results show that the absolute attenuation value has been reduced at high frequencies when the channel length is more than 14 cm.

### 4.2. Long Channel Estimation Results

**Figure 11:**
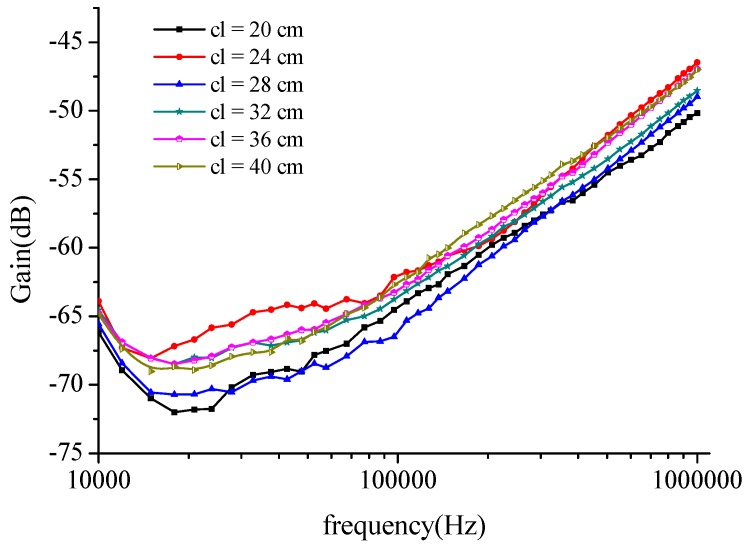
Mean values of the long distance intra-body communication channel characteristic, measured on 10 subjects at different distances.

The measurements for 10 people were repeated several times over a period of days and the average data is reported in Figure 11. The channel length ranged from 20 cm to 40 cm. Voltage attenuation and channel length are seen to be independent of each other, as when the channel length continues to increase, the attenuation remains substantially constant.

It can be seen in Figure 11 that the channel characteristic curves have a slight variation as the signal frequency increases from 10 kHz to 100 kHz, but are almost the same when the frequency is greater than 100 kHz.

**Figure 12:**
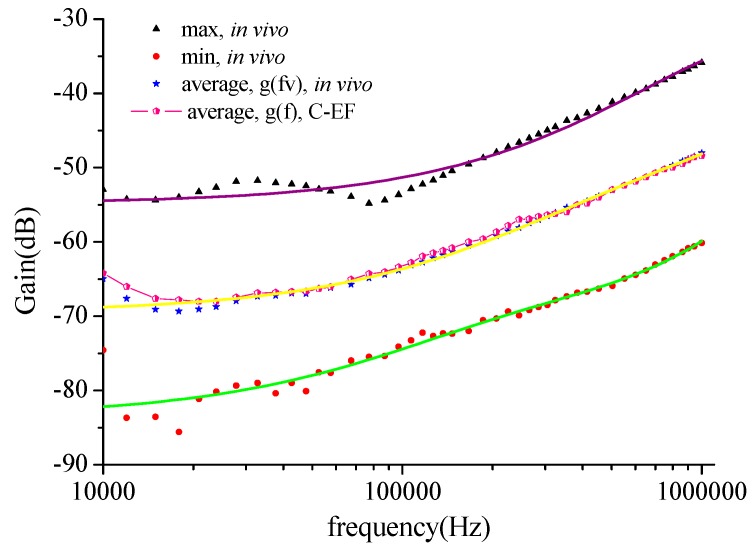
Mean values of the long distance human body channel amplitude characteristic variation range, measured on 10 test subjects. The star curve and red dot line curve represent the average voltage gain for the channel length in the range of 20 cm to 40 cm.

Maximum and minimum *in vivo* measurement results were extracted as the channel characteristics changed in Figure 12. The curve g (*fv*) represents a basic channel characteristics curve for a long length. The trend of the estimation channel characteristics *g*(*f*) agreed with the measured result *g*(*fv*) in Figure 11. Making *G* = *g*(*fv*) + *M*, this denotes the channel characteristics at long distances, where *M* is the correction factor used to correct the gap between the measurement results and estimation results. Generally, *M* is influenced by the individual differences, the measurement precision, the environmental influence, *etc.* From the comparison of estimation results and test results, we found that the correction factor is related to channel length and nearly independent of frequency. This shows that M is a constant in a given channel length regardless of the frequency. For example, the curves of 20 cm and *g*(*fv*) are similar, and the deviation is about constant, and the average error is 1.46 dB. Moreover, a similar phenomenon can also be found when channel length is 32 cm, which shows the errors between the *g*(*fv*) and 32 cm to be 0.079 dB. Therefore, the values of *M* corresponding to the 20 cm and 32 cm lengths were set as 1.46 dB and 0.079 dB. It is important to note that the channel length for different subjects and different experiments will be different.

## 5. Conclusions

This paper employed a field-circuit FEM model of galvanic coupling IBC within an actual measurement environment to realize channel gain estimates based on a field-circuit model. In order to verify the feasibility and the accuracy of the channel characteristic estimation, we carried out measurements. The results indicated that a circuit module equivalent to the external factors which can be added to the field-circuit model, which makes the model more complete, and the estimation based on the proposed field-circuit show better agreeement with the corresponding measurement results.

Specifically, the estimation results based on the proposed field-circuit basically agreed with the corresponding measurement results when the channel length was between 4 cm and 20 cm. The absolute attenuation value of both the estimation results and the *in vivo* measurement results increased as the channel length increased from 4 cm to 20 cm. The peak was found at a frequency of 40 kHz in all cases; this means the best communication frequency of short distance galvanic coupling IBC is in the range of 20 kHz to 60 kHz. The trend of the estimation results also shows better agreement with the measured results in the low frequency range than in the high frequency range, when the channel length is more than 14 cm. The error between measurement results and estimation results is less than 3 dB in the range 10 kHz to 500 kHz, and less than 9 dB in the range 500 kHz to 1 MHz. The error between measurement results and estimation results thus fall within an acceptable range.

When the channel length ranges from 20 cm to 40 cm, the channel characteristics curves have similar outlines, which indicates that there is not a linear relation between channel length and attenuation. The trend of the estimation channel characteristics *g*(*f*) does agree with the measured result *g*(*fv*). Making *G* = *g*(*fv*) + *M*, this denotes the channel characteristics at long distances, where *M* is the correction factor used to correct the gap between the measurement results and the estimation results. Generally, *M* is influenced by the individual differences, the measurement precision, the environmental influence, *etc*.

## Figures and Tables

**Table 1:** A list of acronyms and their meaning.

Shortening	Meaning
cl (cm)	Channel length
EF	Electric Field Model
C-EF	Field-Circuit Model

**Table 2:** The conductivity of tissues at different frequencies.

	σ (s/m)	σ_bone_	σ_fat_	σ_skin_	σ_muscle_x_ = σ_muscle_y_	σ_muscle_z_ = 3σ_muscle_x_
*f* (kHz)						
10		2.043 × 10^−2^	2.383 × 10^−2^	2.041 × 10^−4^	3.408 × 10^−1^	1.022
30		2.057 × 10^−2^	2.412 × 10^−2^	2.293 × 10^−4^	3.475 × 10^−1^	1.043
100		2.079 × 10^−2^	2.441 × 10^−2^	4.513 × 10^−4^	3.619 × 10^−1^	1.086
400		2.177 × 10^−2^	2.477 × 10^−2^	3.048 × 10^−3^	4.278 × 10^−1^	1.283
1000		2.435 × 10^−2^	2.508 × 10^−2^	1.324 × 10^−2^	5.027 × 10^−1^	1.508

**Table 3:** Ratio of characteristic curve and baseline at different distances (in cm).

	*K*(6)	*K*(8)	*K*(10)	*K*(12)
average *K*	1.289	1.498	1.767	2.000
Standard deviation	0.028	0.043	0.058	0.069
